# Cationic micelle-based siRNA delivery for efficient colon cancer gene therapy

**DOI:** 10.1186/s11671-019-2985-z

**Published:** 2019-06-04

**Authors:** Yongping Lu, Lei Zhong, Zhongliang Jiang, Haixia Pan, Yuanfa Zhang, Guonian Zhu, Lan Bai, Rongsheng Tong, Jianyou Shi, Xingmei Duan

**Affiliations:** 1 Pharmacy College, Chengdu university of Traditional Chinese Medicine and The Ministry of Education Key Laboratory of Standardization of Chinese Herbal Medicines of Ministry, State Key Laboratory Breeding Base of Systematic research, development and Utilization of Chinese Medicine Resources, Chengdu, 611137 Sichuan China; 2 Department of Pharmacy, Sichuan Academy of Medical Sciences & Sichuan Provincial People’s Hospital, Personalized Drug Therapy Key Laboratory of Sichuan Province, School of Medicine, University of Electronic Science and Technology of China, Chengdu, 610072 China; 3 Sylvester Comprehensive Cancer Center University of Miami, Miami, USA; 4 Sichuan Academy of Medical Sciences & Sichuan Provincial People’s Hospital, Chengdu, 610072 China; 5 State Key Laboratory of Biotherapy and Cancer Center, West China Hospital, West China Medical School, Sichuan University, Chengdu, People’s Republic of China

**Keywords:** Non-viral vector, Gene therapy, Gene delivery, RNAi, siRNA, C26 colon cancer

## Abstract

Small interfering RNA (siRNA)-based gene therapy has provided an alternative strategy for cancer therapy. One of the key components within gene therapy process is the delivery system. As a novel non-viral gene vector, DMP, prepared by modifying mPEG-PCL micelle with cationic DOTAP lipid, has been prepared and successfully applied in plasmid DNA-based colon cancer gene therapy study. However, its potential in siRNA delivery is unknown. In this study, the preparation process of DMP was optimized and the anti-cancer efficacies of the DMP/siMcl1 and DMP/siBcl-xl complexes were studied on a mouse colon cancer model. Our results demonstrated that DMP cationic micelle-delivered siRNAs could effectively inhibit the growth of C26 colon cancer cells in vitro*.* Meanwhile, intratumoral administration of DMP/siMcl1 and DMP/siBcl-xl complexes obviously suppressed subcutaneous tumor model in vivo. These results suggest the DMP/siRNA complex to be a potential candidate for cancer gene therapy.

## Introduction

Cancer is a major global public health issue. Colon cancer is a common malignant tumor, afflicting over a million people in the worldwide every year [1]. Gene therapy has been applied to serve as a method for treating solid tumors and blood tumors [2, 3]. The key to cancer gene therapy depends on the safe and effective gene delivery system [4, 5]. Non-viral vectors, including cationic lipids, lipid nanoemulsions, solid lipid nanoparticles, and polymer-based vectors have gained more attention than viral vectors for their potential advantages in gene delivery: easily being prepared, causing less immune response, possessing lower toxicity, and better biocompatibility [6, 7]. Nanotechnology provides a novel means for the study of non-viral vectors with advantages including biocompatibility, biodegradability, safety and targeting ability, and so on [2, 8]. Therein, a large number of nanoparticles carriers which have high positive charge can combine with the anionic nucleic acid drug by electric charge effect, showing a wide and bright application prospect in gene therapy [9, 10].

As a type of polymer copolymer, possessing biodegradability and biocompatibility, PEG/PCL copolymers show promising applications in drug delivery systems [11–13]. Based on MPEG-PCL micelles, we used amphiphilic DOTAP to modify MPEG-PCL copolymer in one step, creating the cationic self-assembled DOTAP and MPEG-PCL hybrid micelles (DMP) [14–17]. These micelles show a promising prospect in the delivery of chemical drugs and gene drugs with excellent stability and safety. For example, DMP has been utilized to deliver survivinT34A suicide gene and curcumin for colon cancer therapy [14, 18]. However, previous studies were limited to the transmission of plasmid DNA, and there was no report regarding the delivery of DMP micelles for other forms of gene therapy until now, which limited the application of DMP micelles in gene therapy.

Genetic interference based on siRNA is also an essential part of gene therapy other than therapeutic genes of plasmid DNA. As an essential member of gene-drug, siRNA is a small molecule of double-stranded RNA. siRNA anti-cancer drugs utilize endogenous RNAi mechanisms to silence oncogene expression. Due to significant advantages such as improved stability and silencing efficacy, siRNA has the potential to be developed into cancer therapeutic applications [19]. Compared with the large molecular weight of plasmid DNA, siRNA has higher transfection efficiency, which is particularly suitable for the specific gene-targeting point in gene therapy. Thus, it might be a good choice for gene therapy. Currently, multiple siRNA treatment drugs based on non-viral vectors have been tested in clinical trials, such as CALAA-01 based on cyclodextrin cation polypolymer nanoparticles and ALN-TTR02 based on lipids [2]. However, gene therapy based on siRNA is still needed to develop novel siRNA targeting point and seek safe, effective, and high-specific delivery carriers of siRNA.

In this study, we attempted to use DMP micelles to deliver siRNA to study the efficiency and safety of DMP micelles to deliver siRNA. We used Bcl-xl siRNA and Mcl1 siRNA as a therapeutic target, searching their antitumor effect for treating colon cancer in vitro and in vivo. Bcl-xl gene and Mcl1 gene are members of the Bcl-2 family of the anti-apoptotic gene, and they played a vital role in inhibiting apoptosis [20, 21]. We guess that synthetic DMP micelles can effectively and safely deliver Bcl-xl siRNA and Mcl1 siRNA. Bcl-xl siRNA and Mcl1 siRNA can inhibit Bcl-xl gene and Mcl1 gene expression, inducing apoptosis of C26 cells and then achieving a therapeutic effect of inhibiting the growth of C26 cells.

## Materials and method

### Materials

N-[1-(2,3-dioleoyloxy) propyl]-N, N, N-trimethylammonium methyl sulfate (DOTAP) was purchased from Avanti Polar Lipids (Alabaster, AL, USA). MPEG-PCL diblock copolymer with a designed molecular weight of 4000 was synthesized according to our previous reports [22]. The Mn of MPEG-PCL copolymer was 4050. Dulbecco’s modified Eagle’s medium (DMEM) and 3-(4,5-dimethythiazol-2-yl)-2,5-diphenyl tetrazolium bromide (MTT) were obtained from Sigma-Aldrich (USA) and used without further purification. Dichloromethane and other organic solvents were purchased from ChengDu Kelang Chemical Co., Ltd. (Chengdu, P. R. China). C26 (murine colon adenocarcinoma) cells and 293 T (HEK 293 T/17) cells were purchased from the American Type Culture Collection (Manassas, VA, USA). Cells were cultured in DMEM supplemented with 10% fetal calf serum, incubating at 37 °C with 5% CO_2_-humidified air atmosphere.

### Preparation of DMP micelles

To select a better DMP preparation process, DMP micelles were prepared in three organic solvents, each solvent with three different dosages of DOTAP. Regarding solvent, dichloromethane, chloroform, and ethyl acetate were chosen to study, and the dosages of DOTAP included 5 mg, 10 mg, and 20 mg. DOTAP and MPEG-PCL were co-dissolved in the organic solvent and then evaporated by a vacuum pump to form a dried film. The dried film was subsequently rehydrated in distilled water. Finally, the prepared DMP micelles were stored at 4 °C.

### Characterization of DMP micelles

The particle size and zeta potential of DMP micelles were determined by a Zeta sizer Nano ZS (Malvern Instruments, Worcestershire, UK) and kept at 25 °C during the measuring process. All results were the mean of three runs. The stability of DMP micelles was evaluated qualitatively. Aggregates of DMP micelles were examined by naked eye. The presence of precipitation indicated instability of DMP micelles, while a uniformly transparent solution suggests stability.

### Gel retarding

DMP-delivered Scramble siRNA (DMP/Scramble siRNA) were mixed with 2 μL loading buffer, loaded into 1% agarose gel, and separated by electrophoresis at 140 V for 10 min. 0.2 μg of Scramble siRNA was combined with 1, 2, 3, 4, 5, and 6 μg of DMP micelles. The gel was stained with the gold viewer, and the bands corresponding to Scramble siRNA were visualized under a UV light.

### Cytotoxicity of DMP micelles

The cytotoxicity of DMP micelles on the 293 T cells and C26 cells was evaluated by the MTT method. Briefly, 293T cells and C26 cells were plated at a density of 1.2 × 10^3^ cells per well in 100 μL of DMEM medium containing 10% FBS (fetal bovine serum) in 96-well plate and grown for 24 h. Cells were then exposed to a series of DMP micelles or PEI25K at different concentrations for 72 hours. The viability of cells was measured using an MTT assay. Results were the mean of six test runs.

### In vitro transfection

Twenty-four hours before transfection, C26 cells were seeded into a 24-well plate at a density of 5 × 10^4^ cells per well in 0.5 mL DMEM medium containing 10% FBS (fetal bovine serum). At the time of transfection, the medium in each well was replaced with 0.5 mL medium containing 0%, 10%, 20%, and 30% FBS, and DMP-delivered FAM (Carboxyfluorescein) siRNA (DMP/FAM siRNA) and PEI-delivered FAM siRNA (PEI/FAM siRNA) containing 1 μg of siRNA in serum-free medium were pipetted into each well, and the mass ratio of DMP/FAM siRNA and PEI25K/FAM siRNA were 30:1 and 2:1. Twenty-four hours later, the transferred cells were observed under microscope and luciferase activity was measured by flow cytometry (Epics Elite ESP, USA)

### *In vivo* gene silencing

siRNA-targeting mouse Bcl-xl, Mcl1-1, Scramble siRNA, and FAM-labeled negative control siRNA were purchased from GenePharma Co., Ltd (Shanghai, P. R. China) in unprotected, desalted, annealed form.

To determine the level of Bcl-xl mRNA, total RNA was extracted from C26 cells using TRIzol™ Reagent (Thermo Fisher Scientific, USA), and individual cDNAs were synthesized with a SuperScript II reverse transcriptase assay (Invitrogen). Real-time quantitative PCR was performed with an SYBR GreenER quantitative PCR SuperMix Universal kit (Invitrogen). The PCR primers were synthesized by TSINGKE Biological Technology (Chengdu, P. R. China).

### Anti-proliferation study

The anti-proliferation abilities of DMP/siBcl-xl and DMP/siMcl1 to C26 cells were evaluated by MTT method. Briefly, C26 cells were plated at a density of 1.2 × 10^3^ cells per well in 100 μL of DMEM containing 10% FBS in 96-well plates and grown for 24 h. Cells were then exposed to a series of DMP/siBcl-xl and DMP/siMcl1 at different concentrations for 72 h. The viability of cells was measured using the MTT method. Results were the mean of six test runs.

### Clone formation study

C26 cells were seeded into 6-well plate at a density of 10^3^ cells per well in 2 mL DMEM medium containing 10% FBS. Twenty-four hours later, cells were then exposed to a series of DMP micelles or DMP/Scramble siRNA and DMP/siBcl-xl and DMP/siMcl1 at different concentrations. When the cells grow to a visible clone of the naked eye, the culture was terminated and followed by rinsing, fixing, and dyeing. The number of clones was directly counted, and the inhibition rate was calculated.

### *In vitro* apoptosis study

The cellular apoptosis was observed via Annexin V-fluorescein isothiocyanate (FITC) and propidium iodide staining. Briefly, C26 cells were seeded into six-well plates and exposed to a series of DMP micelles, DMP/Scramble siRNA, DMP/siBcl-xl, and DMP/siMcl1 for 72 hours. Cells were then subjected to Annexin V-FITC and propidium iodide staining.

### In Vivo Tumor Xenograft Study

BALB/c mice of 6–8 weeks old were inoculated with 5 × 10^6^ C26 cells on right flank. DMP/siRNA complexes equivalent to 10 μg of siRNA were injected intratumorally every other day for 5 treatments since the tumor volume reached 100 mm^3^. Mice receiving equivalent amount of normal saline or DMP were regarded as control group. Tumor size was measured and animal weight was monitored every 2 days until all animals were sacrificed. Tumor volume was calculated as (1/2 × length × width [2]).

### Histological analysis

The excised tissues were fixed in 4% neutral-buffered formalin solution for more than 24 hours and embedded in paraffin. Sections of the tissues (3–5 μm) were stained with hematoxylin and eosin. This analysis was performed following the manufacturer’s protocol, and the samples were examined with a fluorescence microscope (× 400).

A commercially available TUNEL kit (Promega, Madison, WI) was used to analyze the apoptotic effects in C26 mouse melanoma xenograft tumor tissues. This analysis was performed following the manufacturer’s protocol.

### Statistical Analysis

Data were expressed as the mean value ± SD. Statistical analysis was performed with one-way analysis of variance (ANOVA) using SPSS software. For all results, *P* ≤ 0.05 was considered to be statistically significant.

## Results

### Preparation process study

To obtain effective transfection efficiency and low cytotoxicity for delivering siRNA, we studied different preparation processes for DMP micelles as shown in Table 1. In our study, regarding solvents, DMP micelles prepared by Ethyl acetate were very unstable, and the stable time of DMP micelles was not more than 10 min. And DMP micelles prepared by dichloromethane and chloroform both had excellent stability, and they could stay stable over 96 hours, except DMP prepared by chloroform with 20-mg dosage of DOTAP. More importantly, they also had the right size, zeta potential, and PDI. However, by comparing initial transfection efficiency, the transfection efficiency of DMP micelles prepared by dichloromethane was higher than that of DMP micelles prepared by chloroform. So we selected dichloromethane to prepare DMP micelles.

**Table 1:** The preparation process of DMP micelles

Sample	Solvent	DOTAP%	Size (nm)	Zeta potential (mV)	PDI	Stability
1	Chloroform	20	80.4	+ 37.7	0.184	24 h
2		10	68.8	+ 25.4	0.233	> 96 h
3		5	83.6	+ 23.6	0.245	> 96 h
4	Dichloromethane	20	144.8	+ 46.4	0.315	> 96 h
5		10	138.7	+ 24.9	0.163	> 96 h
6		5	138.3	+ 20.4	0.153	> 96 h
7	Ethyl acetate	20	/	/	/	<10 min
8		10	/	/	/	<10 min
9		5	/	/	/	<10 min

**Fig. 1:**
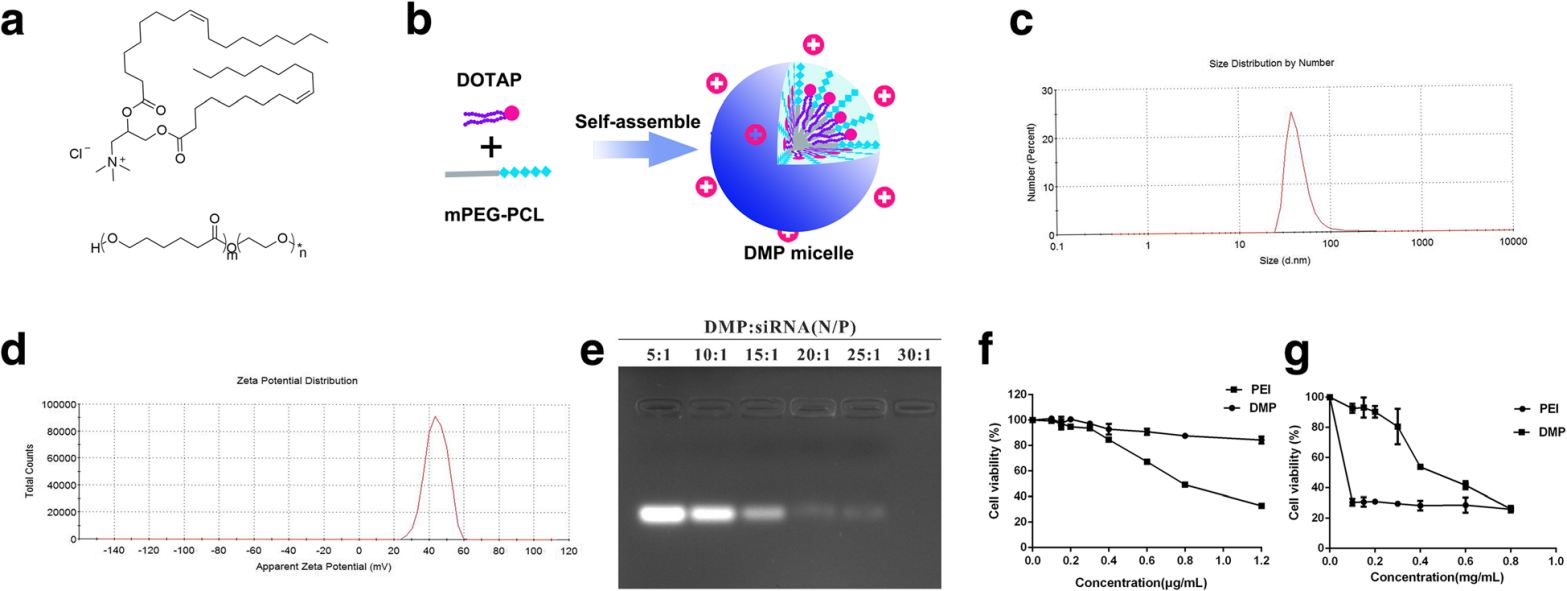
**a** Molecular structure of DOTAP and MPEG-PCL. **b** Preparation scheme of DMP micelles. **c** Size distribution spectrum of DMP micelles. **d** zeta potential spectrum of DMP micelles. **e** siRNA retarding assay. **f** Cytotoxicity of DMP micelles on 293T cells. **g** cytotoxicity of DMP micelles on C26 cells

DMP micelles prepared by three dosages of DOTAP had no apparent differences in size, zeta potential, and PDI, but DMP micelles prepared with a 20-mg dosage of DOTAP had the highest transfection efficiency in three dosages of DOTAP. Through the above comparisons, we selected a preparation process based on DOTAP and MPEG-PCL (Fig. 1a) to prepare DMP micelles with excellent stability and high transfection efficiency. The preparation scheme of DMP micelles is presented in Fig. 1b.

### Characterization of DMP micelles

As presented in Fig. 1c, the particle size distribution spectrum indicated that DMP micelles were nanosized (PDI = 0.315) with a mean particle size of 144.8 nm, suggesting that it had a very narrow particle size distribution. The zeta potential spectrum of DMP was presented in Fig. 1d with a zeta potential of 46.4 mV. The stability of DMP micelles was evaluated qualitatively.

To evaluate the binding ability of DMP with siRNA, a gel retardation assay was performed, and the results were shown in Fig. 1e. When the mass ratio of DMP to siRNA was ≥ 30, complete retardation of Scramble siRNA was achieved. The anionic Scramble siRNA was absorbed on the surface of DMP due to electrostatic interaction, forming a DMP/siRNA complex.

The cytotoxicity of DMP micelles on C26 cells and 293T cells was evaluated by the MTT method. As shown in Fig. 1f and g, PEI25K showed high toxicity on 293T cells, with IC_50_ 0.83 μg/mL. The DMP micelles, however, were much less toxic, and the IC_50_ of DMP was 3.7 μg/mL. The IC_50_ of DMP micelles was 0.497 mg/mL on C26 cells. However, PEI25K showed tremendous toxicity, with IC_50_ < 0.1 μg. Thus, DMP micelles had lower cytotoxicity than PEI25K and could be used to deliver siRNA to C26 cells safely.

### *In vitro* transfection

**Fig. 2:**
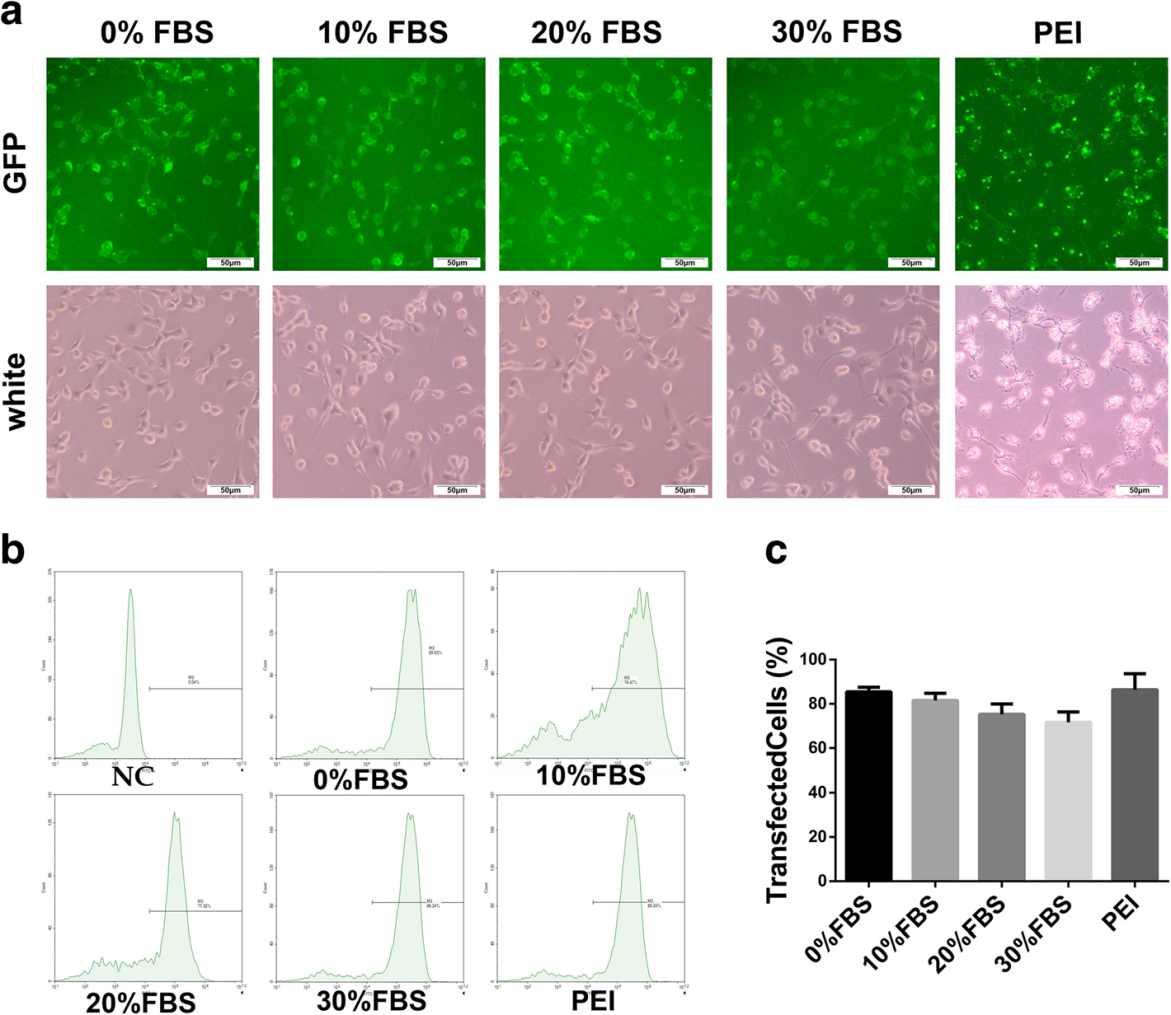
Transfection efficiency of DMP micelles. **a** Picture of transfected C26 cells (**b**, **c**) transfection efficiency of DMP micelles in medium containing 0%, 10%, 20%, and 30% FBS and PEI25K, counted by flow cytometry

Further to biophysical characterization, we compared the transfection efficiency of DMP micelles with that of PEI25K (“gold standard” transfection agent) *in vitro*. As shown in Fig. 2, in medium containing 0%, 10%, 20%, and 30% FBS transfection, DMP micelles had a transfection efficiency of 85.47 ± 1.01%, 81.57 ± 2.04%, 75.29 ± 1.20 %, and 71.64 ± 1.59%, and PEI had a transfection efficacy of 86.38 ± 2.92%. There was little difference in transfection efficiency between serum group and non-serum group. And the transfection efficiencies of serum group and the non-serum group were similar to the transfection efficiency of PEI. Besides, the pictures in Fig. 2a present a direct observation of the transfection ability of DMP micelles with a FAM-based reporter gene. From the picture, the cellar morphology of the PEI group had changed compared with DMP groups, which proved PEI had high cytotoxicity while DMP micelles had low cytotoxicity.

### Anticancer activity *in vitro*

**Fig. 3:**
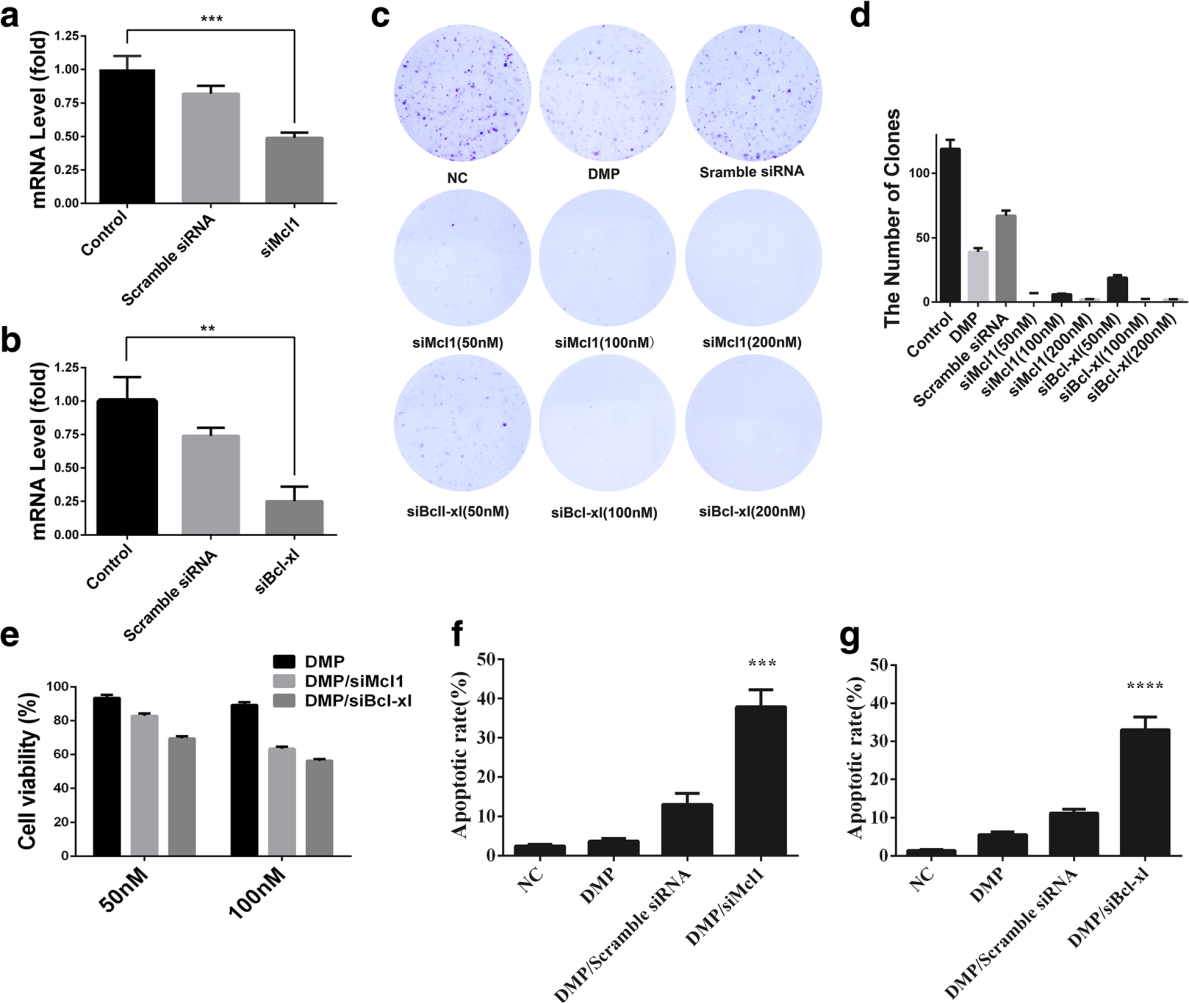
**a** mRNA level of Bcl-xl obviously reduced by DMP/siMcl1. **b** mRNA level of Mcl1 obviously reduced by DMP/siBcl-xl. **c** Pictures of clone formation after treatment with DMP/siBcl-xl or DMP/siMcl1 under different concentrations. **d** The number of clones after treatment with DMP/siBcl-xl of different concentration of siRNA. **e** Anti-cancer ability of DMP/siBcl-xl and DMP/siMcl1with 50-nM and 100-nM concentration of siRNA on C26 cells in vitro. **f** The inhibition rate of clone formation after treatment with DMP/ siMcl1 of different concentration of siMcl1. **g** the inhibition rate of clone formation after treatment with DMP/ siBcl-xl of different concentration of siBcl-xl

In the process of gene therapy, siRNA plays a vital role. Therefore, we designed a Bcl-xl-targeting siRNA and an Mcl1-targeting siRNA to study their anticancer activity. To evaluate the interference ability of Bcl-xl siRNA and Mcl1 siRNA, we confirmed interference ability of Bcl-xl siRNA and Mcl1 siRNA by qPCR. According to our results (Fig. 3), the mRNA level of the DMP/ siMcl1 group was lower than that of the control groups, (Fig. 3a). And the mRNA level of DMP/ siBcl-xl groups was lower than that of the control groups, (Fig. 3b). It has been well demonstrated that DMP/siBcl-xl and DMP/siMCL1 efficiently reduced relevant mRNA level.

Moreover, DMP micelles were used to deliver siRNA into C26 cells to study that DMP/siBcl-xl and DMP/ siMcl1 can inhibit the growth of C26 cells. The viability of the C26 cells was determined by the MTT method. The results showed the cell survival rates of DMP/siBcl-xl (50 nM and 100 nM) were 69.6%±3.3% and 56.3%±1.9%, and cell survival rates of DMP/siMcl1(50 nM and 100 nM) were 82.8%±3.1% and 56.3%±3.2% (Fig. 3e).

For studying further on anticancer activity of DMP/siBcl-xl and DMP/siMcl1, clone formation assay was performed. It was obviously shown that in Fig. 3c and d, compared with the control group, the numbers of clones in DMP/siBcl-xl and DMP/siMcl1 (50 nM and 100 nM) were less. These results suggested that DMP/siBcl-xl and DMP/siMcl1 had an obvious anti-proliferation effect on C26 cells. Thus, combining the results of the MTT assay, the results demonstrated that DMP/siBcl-xl and DMP/siMcl1 could inhibit proliferation of C26 cells and have a prominent anticancer activity on C26 cells.

To determine whether DMP/siBcl-xl- and DMP/siMcl1-induced loss of proliferation capacity and cell viability of DMP micelles were associated with the induction of apoptosis, the numbers of apoptotic cells were assessed by flow cytometry. As shown in Fig. 3f, the apoptosis rates of DMP/ siMcl1 group were 37.9% ± 4.7%, which was higher than that of the control group and DMP group and DMP/Scramble siRNA group. As shown in Fig. 3g, the apoptosis rate of DMP/siBcl-xl group was 33.0% ± 3.8%, which was also higher than that of other control groups. These apoptosis data indicated that DMP/ siMcl1 and DMP/ siBcl-xl had a strong apoptosis-inducing ability. The apoptosis assay results were consistent with the MTT results, and clone formation results described earlier, suggesting that inducing apoptosis is a possible mechanism for inhibiting the proliferation and viability of C26 cells.

### DMP/siRNA complex inhibit C26 tumor growth *in vivo*

**Fig. 4:**
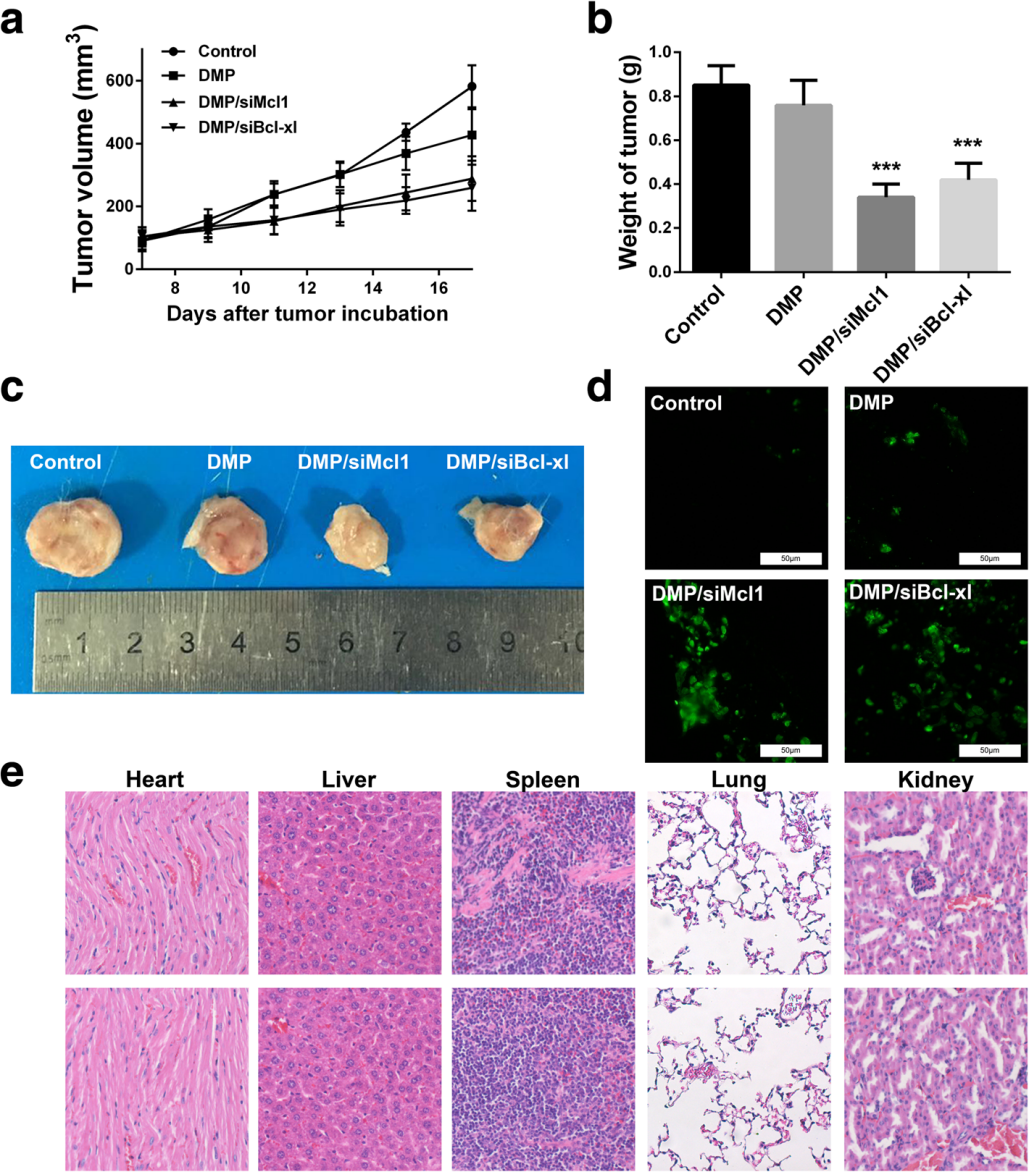
Anticancer effects and safety of DMP/siRNA on C26 mouse xenograft model. **a** Tumor development curve, calculated by tumor volume. **b** Average weight of tumors in each group. Compared with other treatment groups, the DMP/siMcl1 group and DMP/ siBcl-xl group achieved a statistically significant reduction in tumor weight (***P, 0.001). **c** Representative images of tumors of C26 colon carcinoma. **d** Apoptosis in tumor tissues detected by TUNEL assay. **e** HE analysis of main organs from each treatment group in both models. No significantly pathological changes were observed in heart, liver, spleen, lung, or kidney

A C26 xenograft animal model was also utilized to test the antitumor efficacy of DMP/siRNA complex in vivo. The tumor growth curves and images of C26 xenograft tumors of each group are presented in Fig. 4a and b. According to our results, intratumoral injection of DMP/siMcl1 and DMP/siBcl-xl resulted in a significant inhibition of xenograft tumor growth compared with control groups. The weight of the tumors in each group is presented in Fig. 4b. Comparing with NS treatment group (0.85 ± 0.09 g) and DMP group (0.76 ± 0.11 g), DMP/siMcl1 complex caused a statistically significant reduction in tumor weight (0.34 ± 0.06 g, *P* < 0.01), and meanwhile, DMP/siBcl-xl complex caused a statistically significant reduction in tumor weight (0.42 ± 0.08 g, *P* < 0.01). These results suggest that intratumoral injection of DMP/siRNA complex could efficiently inhibit the growth of subcutaneous xenograft of C26 colon cancer model. The in vivo side effects of DMP/siRNA complex on other organs were examined through HE analysis. As shown in Fig. 4, no significantly pathological changes in the heart, liver, spleen, lung, or kidney were observed.

## Discussion

In this study, cationic self-assembled DOTAP and MPEG-PCL hybrid micelles were prepared to deliver Bcl-xl siRNA and Mcl1 siRNA for treating C26 colon cancer. These DMP micelles had excellent stability and were able to effectively combine and transmit Bcl-xl siRNA and Mcl1 siRNA to C26 cells. More importantly, DMP micelles unprecedentedly delivered siRNA (Bcl-xl siRNA and Mcl1 siRNA) and could effectively induce the apoptosis of C26 cells, resulting in inhibiting the growth of C26 cells both in vitro and in vivo with high safety. Collectively, our study suggested that DMP is a potential gene delivery carrier for siRNA.

There are reports of that cationic lipid DOTAP have been widely applied for delivery of siRNA, but it engenders strong positive charges triggering hemolysis and had high cytotoxicity. Based on DOTAP cationic liposomes with high transfection efficiency, but their positive charges would produce toxicity, the reason may be that quaternary ammonium salt head of DMP with positive charges was exposed on the surface of the liposome phospholipid bilayer. However, DMP micelles based on DOTAP lipid had high transfection and low cytotoxicity. We speculated that in micelles self-assembled process, DOTAP was embedded inside the polymer nanoparticles, of which quaternary ammonium salt head was not completely exposed on the surface. Thus the partial positive charges were masked; in the meantime, our results also show that the transfection efficiency of DMP micelles was not low compared with the transfection efficiency of PEI25K. It suggested that the positive charges of DOTAP after being covered were still partially exposed, showing proper potential, and this level of potential was proved to be sufficient to combine and transmit siRNA. Furthermore, our results indicated that DMP micelles based on MPEG-PCL is an effective and safe gene delivery carrier, which has been optimized for the cytotoxicity of DOTAP and retains the gene transmission capacity of DOTAP. And a similar study had reported that the cytotoxicity of DOTAP was reduced after being combined with other materials [23]. Recently a novel vector consisting of DOTAP and SWNT had lower cytotoxicity in siRNA delivery compared with lipofectamine [8]. LPNs which are prepared DOTAP-PLGA micelles by using a double-emulsion solvent evaporation (DESE) method resulted in nano-sized carriers [24–26]. These studies agreed well with that described in our research and made our conjecture more convincing to a certain extent.

The delivery efficiency of the gene transfer vector is highly influenced by serum. The serum proteins in the serum could neutralize the positive charges on the surface of the nanocarrier/siRNA complexes, which was affected by the electrostatic effects on the surface of cells with negative charges [27–29].

In our study, when we studied the transfection efficiency of DMP micelles, we set up a series of DMEM medium containing 0%, 10%, 20%, and 30% FBS for transfection, investigating the effect of serum on the transfection efficiency of loaded FAM siRNA nanoparticles. Our results showed the presence of FBS had little impact on transfection efficiency. In this article, we used MPEG-PCL to cover DOTAP, and DOTAP was embedded inside MP with some parts left on the surface in a proper proportion. Based on this structure, we speculated that the positive charges of the DOTAP were partially hidden due to the cover of MPEG-PCL, which decreased the level of exposing to serum proteins, so the effect of serum on transfection was avoided. It implied that besides the reduced surface charges of cationic carriers after modification, the presence of serum transfection had little effect on the efficiency of the cationic polymer carrier. Due to the excellent transfection capacity in serum environment demonstrated by DMP micelles in vitro, it suggested that the serum had little affection for DMP-mediated siRNA delivery in vitro cell experiment.

## Conclusion

In this study, the prepared DMP micelles had the capacity for delivering siRNA. And DMP micelles were used to deliver Bcl-xl siRNA and Mcl1 siRNA into C26 cells for anti-cancer activity study in vitro. We investigated the preparation process for DMP micelles and selected an excellent preparation process to prepare DMP micelles with narrow size, good zeta potential, and excellent stability. Besides, the prepared DMP micelles were not affected by the presence of serum in vitro transfection experiment. More importantly, DMP/siBcl-xl and DMP/siMcl1 can inhibit Bcl-xl gene and Mcl1 gene expression, inducing apoptosis of C26 cells and then achieving a therapeutic effect of inhibiting the growth of C26 cells. Intratumoral injection of DMP/siRNA complex could efficiently inhibit the growth of subcutaneous xenograft of C26 colon cancer model. No significantly pathological changes in the heart, liver, spleen, lung, or kidney were observed. It is believed that DMP micelles can be considered as an effective carrier to deliver siRNA for treating colon cancer.

## Abbreviations

DOTAP: N-[1-(2, 3-dioleoyloxy) propyl]-N, N, N-trimethylammonium methyl sulfate
mPEG-PCL: Methoxypoly (ethylene glycol) Poly (caprolactone)
MTT: 3-(4, 5-dimethythiazol-2-yl)-2,5-diphenyl tetrazolium bromide
siRNA: Small interfering RNA
